# Sun exposure and challenges of sun protection in adolescents and young adults: A prevalence observational study

**DOI:** 10.3892/mi.2024.195

**Published:** 2024-10-07

**Authors:** Andre Friedli, Evelyne Fournier, Thomas Haynes Hutson, Emilien Jeannot

**Affiliations:** 1 Department of Dermatology, Clinique Hirslanden des Grangettes, 1224 Chêne-Bougeries, Switzerland; 2 Geneva Cancer Registry, University of Geneva, 1200 Geneva, Switzerland; 3 Wyss Center for Bio and Neuroengineering, 1202 Geneva, Switzerland; 4 Institute of Global Health, Faculty of Medicine, University of Geneva, 1202 Geneva, Switzerland; 5 Community Psychiatric Service, Lausanne University Hospital, 1011 Lausanne, Switzerland

**Keywords:** skin protection, sun exposure, sun protection, sunburn prevention, adolescent

## Abstract

The present study aimed to explore the adherence to and efficiency of skin protection measures among teenagers and young adults. The present study investigated the reasons for sun exposure and the obstacles impeding sun protection. In the summer of 2023, a self-reported questionnaire was posted on the social media account of two high schools, a university, some industrial companies and among the teenage children of a dermatology clinic. Descriptive statistics and multivariate logistic regression analyses were performed to assess the reasons for sun exposure behavior, sun protection measures and the frequency of sunburn among individuals aged 15 to 25 year. A total of 517 young individuals completed the questionnaire. It was found that 43.5% use a hat as a means of sun protection, and only 10% try to limit their exposure to the sun. Nevertheless, 78.7% indicate the use of sun cream, 61.7% expose themselves frequently or very frequently to the sun, and 51.2% exposed themselves to the sun for tanning. Young females were statistically more prone to tan [odds ratio (OR), 2.95; 95% confidence interval (CI), 1.95-4.46] P=0.03 than males. Males exposed themselves to a 3-fold greater extent during recreational activities (OR, 2.98; 95% CI, 1.56-5.69) P=0,025, compared to females. On the whole, the present study demonstrates that young individuals aged 15 to 25 years are exposing themselves too frequently to the sun. A large proportion of individuals still wishes to acquire a tan. Even if the large majority of individuals uses sunscreen, the majority of these do not protect themselves correctly since they report becoming sunburnt frequently.

## Introduction

The incidence of melanoma (MM) and non-melanoma skin cancer (NMSC) is steadily on the rise worldwide with an increasing impact on public health (1). Switzerland has one of the highest skin cancer rates worldwide (https://derma.plus/globaler-hautkrebs-index) with MM being the fourth most common type of cancer among males and females in Switzerland (https://www.bfs.admin.ch/bfs/de/home/statistiken/gesundheit/gesundheitszustand/krankheiten/krebs.html). The main known risk factor for skin cancers is exposure to UV-light (2). Primary prevention through sunscreen, protective clothing, hats and using shaded areas could reduce the incidence of all types of skin cancer (3). Sunburn and intense exposure to the sun among children and young adults is particularly hazardous (4). A genetic causal association between childhood sunburn and the risk of developing both MM and NMSC was recently found, which suggested that the prevention of childhood sunburn could contribute to the decreased risk of developing MM and NMSC later on in life (5). Various educational programs targeting young individuals are undertaken in Switzerland and elsewhere (https://www.krebsliga.ch/ueber-krebs/praevention/vor-der-sonne-schuetzen/sonnenschutz-fuer-kinder) (6). However, sun protection is a low-priority issue for adolescents (7) and they are significantly less likely than adults to engage in sun protection practices (8).

A decade ago, sun protective behavior in North-Western Switzerland was insufficient and 60% of individuals experienced sunburn over the preceding year (9). Of note, 69.2% of schoolchildren reported applying sunscreen routinely; however, 25% did not use sunscreen as they preferred to acquire a tan, and 11% did not use sunscreen as they did disliked the texture of the products. Another study demonstrated that in Switzerland, the percentage of young individuals with high-level knowledge of sun protection was lower than that in other countries with a lower incidence of skin cancer (10).

A previous study demonstrated that tanning and outdoor sport activities were the main reasons for sunburn among college students (11). Tanning continues to be very popular worldwide. The TikTok tag #tanlines has 430 million followers and in Switzerland, 8% of the population continues to use tanning devices (https://www.bag.admin.ch/bag/de/home/gesund-leben/umwelt-und-gesundheit/strahlung-radioaktivitaet-schall/elektromagnetische-felder-emf-uv-laser-licht/solarium.html).

The present observational study aimed to explore the adherence to skin protection measures among teenagers and young adults in western Switzerland. The reasons for sun exposure and the challenges impeding sun protection were investigated. The present study also investigated whether the skin cancer prevention programs of the past decade reached adolescents and young adults, as well as whether this group in western Switzerland understands the link between sunburn and skin cancer. In addition, the present study examined the type of sun protection the participants applied, the reasons for not applying protection and whether this protection was sufficient to avoid sunburn.

To the best of our knowledge, these questions have not been asked for 10 years in the greater Geneva region and according to the answers obtained, the quality of skin cancer prevention may have to be adapted in order to better explain the harmful effects of exposure to UV-light, as well as protection measures.

In the summer of 2023, a skin cancer prevention campaign was performed in Geneva and western Switzerland. Humoristic cartoons warning of the dangers of the sun were distributed over multiple social media accounts, targeting young individuals aged 15 to 25 years (https://vimeo.com/910238062). As part of the evaluation process, a survey was conducted to determine what part of the target population had viewed the campaign. With the same questionnaire, the reasons for sun exposure, adherence to sun protection and the incidence of sunburn were investigated. The results obtained are described in the present scientific study.

## Subjects and methods

### Study population

The study population consisted of young individuals, aged 15 to 25 years, residing in the Geneva region (n=65,000 people, 12% of the total population of the Geneva region). Sunburn in this age group and in younger individuals is particularly hazardous. This survey was based on an anonymous self-reported questionnaire. The study period was 2 months, between June 15 until August 15, 2023.

### Sample size

The minimum sample size (number of included questionnaires) was calculated as 382 with open EpiData (population size, 65,000; anticipated frequency, 50%; absolute precision, 5%; design effect, 1.0). The present study assumed subgroups of similar sizes and that ~50% of the participants would have the outcome (sunburn or sun protective measures).

### Questionnaire

A digital questionnaire was created on Google Docs. This questionnaire was available as a QR-code, or as a web-link and contained multiple choice questions only. No special pretesting of the questions has been done. Certain standard outcome measures of sun exposure and sun protection habits could be included (12). To define educational level, the participant could select between university student, high school student or no higher studies/apprentice. The other questions focused on the reasons for exposure, last sunburn event, adherence to protective measures (sunscreen, clothing, hat and shade) and obstacles for using sunscreen. No further specifications of sunscreen use (always, often, sporadic) were asked.

The questionnaire (Data S1) was posted at the social media account (Instagram) of two high schools in Geneva. Those schools published a short Instagram story about the survey. Human resources officers of several companies [Rolex, Geneva University Hospital (HUG), Fédération des Entreprises Romandes Genève (FER) and others] were asked to propose the questionnaire to their apprentices and young workers, in order to involve participants with a lower education background.

### Ethics approval

The present study was approved by the Institutional Review Board of Geneva county ethics. (Req-2023-01182) and was performed in accordance with the principles of the Declaration of Helsinki. Online written informed consent was obtained for the publication of this study.

### Statistical analysis

Data are presented as absolute numbers and percentages. A multivariate logistic regression analysis was performed separately for each outcome. The considered outcomes were the following: Frequent exposure to the sun, reasons for exposure (tanning, sport, no exposure), measures of protection (hat, sunscreen, shade, protection garments, no exposure, no protection) and sunburn. Associations between outcomes and explanatory variables were presented in the form of odds ratios (ORs). All statistical analyses were performed using Stata 18 (StataCorp LP, College Station, Texas, USA). A value of P<0.05 was considered to indicate a statistically significant difference.

## Results

### Participants

A total of 566 young individuals completed the questionnaire between June 20 of until the end of August, 2023; 80% of all participants were enrolled within the first 3 weeks. Of note, 49 participants were excluded as they were either <15 years or >25 years of age. In total, 517 answers were analyzed. Of the participants, two thirds (328/517) were female; one third of the participants was >20 years old, and the remaining 70.6% were younger. As regards the distribution of responses according to their level of education, it was observed that 111 (21.5%) were in apprenticeship, 208 (40.2%) at university and the remaining 198 (38.3%) were still going to school (Table I). This occupational distribution corresponds to the population of this age-group in Geneva.

### Sun protection habits

The sun protection habits of the study population are shown in Table II. It can be observed that 88 persons (17%) do not use any sun protection (did not use sunscreen or covering garments or a hat or do not look for shade or do not limit exposure). Of the study population, 12.9% wear special clothing, 43,5% wear a hat, 51% seek shade, 78.7% use sunscreen-In addition, 10.6% stated they limit their exposure to the sun as a means of protection. It is also worth noting that, whatever the means of protection used, younger teenagers, girls and those with a higher level of education reported using them in greater numbers than older teenagers/young males, males and the population with a lower level of education.

As demonstrated in Table III, multivariate analysis revealed that males and individuals with a lower level of education use less sunscreen [OR, 0.51; 95% confidence interval (CI), 0.32-0.82; P=0.01; and OR, 0.40; 95% CI, 0.22-0.74, P=0.01 respectively]. It was also observed that apprentices wear less special clothing (OR, 0.22; 95% CI, 0.09-0.54) P=0.005, or a hat (OR, 0.45; 95% CI, 0.27-0.76) P=0.015 compared with university students.

### Sun exposure

Sun exposure frequency and the reasons for sun exposure are presented in Table IV. It was noted that 61.7% of young adults and adolescents in Geneva declared to expose themselves frequently or very frequently to the sun. Half of them (51.2%) exposed themselves to the sun for tanning purposes. A total of 191 individuals (36.9%) believed that exposure to the sun was good for their health.

In terms of their knowledge of sunburn and methods of protection, 35. 8% indicate they do not have sufficient information and want more information on the dangers of the sun. A further, 40.8% declare they have this information, but still need more. On the other hand, 19.5% stated that they receive sufficient information about the dangers of the sun. This is in line with the fact that almost 40% stated that they voluntarily expose themselves to the sun as they consider that it is beneficial for their health.

As shown in Table V, males and females exposed themselves to the sun for various reasons: Males exposed themselves at 3-fold greater level during recreational activities (OR, 2.98; 95% CI, 1.56-5.69; P=0.025), compared to females. In addition, females were statistically 3-fold more prone to tan (OR, 2.95; 95% CI, 1.95-4.46; P=0.03) than males.

### Frequency of sunburn

The reported frequency of sunburn in this adolescent/young adult population is presented in Table VI. It was noted that 37.5% of the investigated adolescents and young adults burn continuously during the last summer, while another 33% suffered their last sunburn within the past year. It was observed that girls reported having been burnt continuously more often than boys, and young teenagers also reported having sunburned more often than the older population.

## Discussion

The majority of young individuals in Geneva expose themselves to the sun frequently or even very frequently. Half of them do this for the purpose of acquiring a tan, particularly young females and individuals with a lower level of education. The majority of them suffer from sunburn while doing so. Previous research has already demonstrated this tendency among college students (12). The population in the present study was older and from a metropolitan region, and exhibited the same trends, if not worse. Subgroup analysis revealed a tendency for older individuals and university students to be more reasonable, to protect themselves more effectively and to suffer less from sunburn.

Overall, the prevalence of sunburn in the present study was very high. Of note, 87% of the participants with repeated sunburn reported the use of sunscreen. However, the survey performed demonstrated that this form of sun protection or the way in which it is applied by the study population, is not an effective method to prevent sunburn. This is in contrast to the findings of other studies, where sunscreen, applied correctly, was one of the most effective methods for reducing skin cancer (13). One reason for this failure is that the sun protection factor (SPF) is a confusing label and is often not fully understood (14). It is already known, that intentional and unintentional sun exposure with sunscreen may increase the duration of exposure with an increased risk of sunburn (15,16). Educational efforts are necessary to explain to young individuals the limits and practical use of sunscreen and that frequent exposure should not be performed with this protection only. The issue is that shade, hats and special clothing are even less popular measures. In the present study, even the participants who suffered from repeated sunburn do not limit their exposure, but continued to use sunscreen only. It remains to be determined whether this is due to a knowledge-action gap or a simple lack of education.

Indeed, two thirds of the present study population would welcome more information on skin cancer and prevention measures. Young individuals need to understand the link between exposure to UV-light and skin cancer. In Geneva, for example, the youth health service of the public education department organizes courses and prevention campaigns in schools at various stages of their pupils' education, to inform them of the risks of melanoma linked to exposure to the sun (https://www.ge.ch/document/promotion-sante-prevention-dans-structures-accueil-petite-enfance). The various university hospitals in Switzerland are also deeply involved in screening and raising awareness of this problem among young individuals and teenagers. However, these initiatives are only cantonal, and there is no concerted, organized national policy in Switzerland to ensure that all pupils have access to these health education programs.

The importance of avoidance strategies, clothing and seeking shade should be the first line in future projects. Skin cancer prevention campaigns should include new approaches, encouraging intention and habits (17) to better target the knowledge action gap and behavioral changes. Prevention measures and messages need to be adapted to the development of cognitive functions of adolescents. Messages should be formulated as concretely as possible, and focus on immediate and important effects on the present lives of adolescents. For example, for smoking prevention, it is more effective to allow a young individual calculate the financial cost of his consumption compared with his pocket money, than to talk to him about the dangers of future lung cancer. It would be interesting to observe how these strategies could be adapted for the prevention of melanoma.

The design of prevention measures should be guided by development tasks. The aim is to create spaces where adolescents can explore and train their physical, cognitive, mental and social skills through play. They need to be able to experience their own efficacy through the achievement of personal goals, but also through the recognition of their contribution to family and society.

The present study had certain limitations which should be mentioned. The data presented reflect the conditions of 15 to 25-year-old individuals, living in greater Geneva. The numbers may be similar in other parts of the country, although they could also differ. In Switzerland, there is no official national program in schools for skin cancer prevention. Each county (Canton) has different prevention programs, mostly initiated by private and non-governmental associations. Similar study populations in other parts of the country could be better or less informed.

In the survey performed herein, pragmatic sampling we used. It is possible, that the questionnaire was distributed more in parts of the city and surroundings with higher-than-average income and higher education. Therefore, there may have been an underrepresentation of individuals with a lower level of education. Since these individuals have a reduced tendency to follow the protective guidelines, the actual incidence of sunburn could be more severe. Furthermore, since the questionnaires were self-administered via an online questionnaire, there were certainly some false answers due to a misunderstanding of the questionnaire.

In conclusion, the present study demonstrates that in western Switzerland, young individuals between aged 15 and 25 years are exposing themselves to the sun too often and without sufficient sun protection, and thus experience sunburn frequently. Educational efforts are necessary to explain the limits of sunscreen use and encourage other methods of sun protection.

## Supplementary Material

Questionnaire of the study.

## Figures and Tables

**Table I:** Demographic characteristics of the study participants.

Characteristic	No. of participants	%
Age, years		
15-20	365	70.6
21-25	152	29.4
Total	517	100.0
Sex		
Female	328	63.4
Male	181	35.0
Other^a^	8	1.5
Education level		
No superior studies/apprentice	111	21.5
High school	198	38.3
University student	208	40.2

^a^Other indicates that the female or male sex was not checked in the questionnaire, but instead the box ‘other’ was checked.

**Table II:** Sun protection habits.

A, Sun protection, I use covering garments						
	No (n= 450; 87.1%)		Yes (n=67; 12.9 %)		Total (n=517)	
Characteristic	No. of participants	%	No. of participants	%	No. of participants	%
Age, years						
15-20	326	63	39	7.5	365	70.5
21-25	124	23.9	28	5.4	152	29.6
Sex						
Female	289	55.8	39	7.5	328	63.4
Male	156	30.1	25	25	181	36.6
Other^a^	5	0.9	3	4.8	8	
Education level						
No superior studies/apprentice	104	20.1	7	1.35	111	21.4
High/middle school	178	34.4	20	3.8	198	38.3
University student	168	32.4	40	7.7	208	403
B, Sun protection, I use a hat						
	No (n=292; 56,5%)		Yes (n=225;43.5%)		Total (n=517)	
Characteristic	No. of participants	%	No. of participants	%	No. of participants	%
Age, years						
15-20	223	43.1	142	27.4	365	70.5
21-25	69	13.3	83	16	152	29.5
Sex						
Female	190	36.7	138	26.7	328	63.4
Male	101	19.5	80	15.5	181	35
Other^a^	1	0.19	7	1.35	8	1.6
Education level						
No superior studies/apprentice	70	13.5	41	9	111	21.4
High/middle school	132	25.5	66	12.7	198	38.3
University student	90	17.4	118	22.8	208	40.2
C, Sun protection, I search for shade						
	No (n=253; 49%)		Yes (n=264; 51%)		Total (n=517)	
Characteristic	No. of participants	%	No. of participants	%	No. of participants	%
Age, years						
15-20	195	37.7	170	32.8	365	70.6
21-25	58	11.2	94	18.1	152	29.4
Sex						
Female	160	30.9	168	32.4	328	63.4
Male	91	17.6	90	17.4	181	35
Other^a^	2	0.3	6	1.2	8	1.5
Education level						
No superior studies/apprentice	63	63	48	9.3	111	21.4
High/middle school	104	20.1	94	18.2	198	38.3
University student	86	16.6	122	23.6	208	40.3
D, Sun protection, I use sunscreen						
	No (n=110;21.3%)		Yes (n=407; 78.7%)		Total (n=517)	
Characteristic	No. of participants	%	No. of participants	%	No. of participants	%
Age, years						
15-20	76	14.7	289	71	365	70.6
21-25	34	0.06	118	22.8	152	29.4
Sex						
Female	275	53.2	38	7.35	333	64.4
Male	152	29.4	22	4.2	174	33.6
Other^a^	7	1.35	3	0.58	10	2
Education level						
No superior studies/apprentice	95	18.3	40	7.7	135	26.1
High/middle school	155	29.9	27	5.2	182	35.2
University student	170	32.8	30	5.8	200	38.7
E, Sun protection, I limit exposure						
	No (n=462; 89.4 %)		Yes (n=55; 10.6%)		Total (n=517)	
Characteristic	No. of participants	%	No. of participants	%	No. of participants	%
Age, years						
15-20	334	64.6	31	5.9	365	70.6
21-25	128	24.7	24	4.6	152	29.4
Sex						
Female	290	56	38	7.35	328	63.4
Male	166	32	15	2.9	181	35
Other^a^	6	1.2	2	0.38	8	1.6
Education level						
No superior studies/apprentice	106	20.5	5	0.9	111	21.5
High/middle school	177	34.2	21	4	198	38.3
University student	179	34.6	29	5.6	208	40.2

^a^Other indicates that the female or male sex was not checked in the questionnaire, but instead the box ‘other’ was checked.

**Table III:** Multivariate analysis of the sun protection habits.

A, I use sunscreen to protect myself from sunburn				
Parameter	OR	95% CI min	95% CI max	P-value
Age, years				
<20	Reference	-	-	-
21-25	0.78	0.48	1.26	NS
Sex				
Female	Reference	-	-	
Male	0.51	0.32	0.82	0.01
Education level				
No superior studies/apprentice	0.40	0.22	0.74	0.01
School (high school/middle school)	1.54	0.93	2.53	NS
University student	Reference	-	-	-
B, I use covering garments to protect myself from sunburn				
Parameter	OR	95% CI min	95% CI max	P-values
Age, years				
<20	Reference	-	-	-
21-25	0.91	0.69	1.37	NS
Sex				
Female	Reference	-	-	-
Male	0.81	0,51	1.02	NS
Education level				
No superior studies/apprentice	0.22	0.09	0.54	0.005
School (high school/middle school)	1.25	0.92	1.58	NS
University student	Reference	-	-	-
C, I use a hat to protect myself from sunburn				
Parameter	OR	95% CI min	95% CI max	
Age, years				
<20	Reference	-	-	-
21-25	0.85	0.33	1.43	NS
Sex				
Female	Reference	-	-	-
Male	0.76	0.45	1.07	NS
Education level				
No superior studies/apprentice	0.45	0.27	0.74	0.015
School (high school/middle school)	1.05	0.85	1.25	NS
University student	Reference	-	-	-

NS, no statistically significant difference.

**Table IV:** Exposure frequency of the sun and reason of exposition.

Exposure frequency	No. of participants	%
Very rarely/rarely	31	6.00
Sometimes	167	32.3
Frequently/very frequently	319	61.7
I expose myself to the sun, during recreational activities		
No	77	14.9
Yes	440	85.1
I expose myself to the sun, because I want to tan		
No		48.7
Yes		51.2
I expose myself to the sun, because this is good for my health		
No	326	63.1
Yes	191	36.9
Sun protection, I do not protect myself against the sun		
No	429	83.00
Yes	88	17.00
I do not use sunscreen because I want to tan		
No	412	79.7
Yes	105	20.3
I do not use sunscreen because I do not like the texture, color…		
No	406	79.7
Yes	111	20.3
I do not use sunscreen because I am afraid of the ingredients		
No	481	93.00
Yes	36	7.00
I do not use sunscreen because I want to protect the environment		
No	492	95.2
Yes	25	4.8
Is there sufficient information about the dangers of the sun		
Yes, we are very well informed	101	19.5
There is information but not a lot	211	40.8
Not enough information, we need more	185	35.8
I do not want more information	20	3.9

**Table V:** Multivariate analysis of reasons for sun exposure.

A, I expose myself to the sun, during recreational activities				
Parameter	OR	95% CI min	95% CI max	P-values
Age, years				
<20	Reference	-	-	-
21-25	0.98	0.68	1.46	NS
Sex				
Female	Reference	-	-	-
Male	2.98	1.56	5.69	P=0.025
Education level				
No superior studies/apprentice	0.90	0.52	1.42	NS
School (high school/middle school)	1.45	0.93	2.01	NS
University student	Reference	-	-	-
B, I expose myself to the sun, because I wish to tan				
Parameter	OR	95% CI min	95% CI max	
Age, years				
<20	Reference	-	-	-
21-25	0.91	0.69	1.37	NS
Sex				
Female	2.95	1.95	4.46	P=0.03
Male	Reference	-	-	-
Education level				
No superior studies/apprentice	1.45	0.99	1.46	NS
School (high school/middle school)	1.28	0.90	1.66	NS
University student	Reference	-	-	-
NS, no statistically significant difference.				

**Table VI:** Frequency of sunburn

Parameter	I never have sunburn		Several years prior		Within the last year		This summer, I burn continuously	
Variables	N	%	N	%	N	%	N	%
Age, years								
15-20	51	9.8	56	10.8	120	23.2	138	26.6
21-25	19	3,7	26	5	51	9.8	56	10.8
Total	70	13.5	82	15.8	171	33	194	37.5
Sex								
Female	43	8,3	53	10.2	107	20.6	125	24.1
Male	27	5.2	29	5.6	58	11.2	67	12.9
Other^a^	0	0.0	0	0.0	6	1.2	2	0.4
Education level								
No superior studies/apprentice	23	4.4	18	3.4	32	6.2	38	7.3
School (highs/middle school)	25	4.8	27	5.2	64	12.3	82	15.8
University student	22	4.2	37	7.1	75	14.5	74	14.3

^a^Other indicates that the female or male sex was not checked in the questionnaire, but instead the box ‘other’ was checked.

## Data Availability

The datasets used and/or analyzed during the current study are available from the corresponding author on reasonable request.
