# Supplementary material for: Sun exposure and challenges of sun protection in adolescents and young adults: A prevalence observational study
Source: Med Int (Lond). 2024 Oct 7;4(6):71. doi: 10.3892/mi.2024.195 (PMC11485268; doi:10.3892/mi.2024.195)
Supplement: Questionnaire of the study. [file Supplementary_Data.pdf]

Data S1. Questionnaire of the study.

YOUNG PEOPLE AND THE SUN

1. Your age:
- ☐ Less than 15
- ☐ 15 – 19
- ☐ 20 – 25
- ☐ More than 25

2. Gender?
- ☐ Male
- ☐ Female
- ☐ Other

3. Education:
- ☐ School (high school/middle school)
- ☐ Apprentice
- ☐ University-student
- ☐ No superior studies

4. Sun exposure:
- ☐ Very frequently
- ☐ Frequently
- ☐ Sometimes
- ☐ Rarely
- ☐ Very rarely

5. I expose myself to the sun
(multiple answers possible)
- ☐ During recreational activities (sport, swimming)
- ☐ Because I want to tan
- ☐ Because it is good for my health (vitamin D)
- ☐ As little as possible

6. Sun protection: I protect myself
(multiple answers possible)
- ☐ I limit exposure
- ☐ I search for shade
- ☐ I use covering garments
- ☐ Use a hat
- ☐ Use sunscreen
- ☐ I don't protect myself against the sun

7. I don't use sunscreen because
(multiple answers possible)
- ☐ I want to tan
- ☐ I don't like the texture, color…
- ☐ I'm scared of the ingredients
- ☐ I want to protect the environment
- ☐ No, indeed, I use sunscreen

8. Sunburn: When was the last?
- ☐ I never have sunburn
- ☐ Several years ago
- ☐ Within the last year
- ☐ This summer, I burn all the time

9. Have you seen the stopskincancer clip?

- ☐ Yes
- ☐ No

10. Is there sufficient information about the dangers of the sun

- ☐ Yes, we are very well informed
- ☐ There is information but not a lot
- ☐ No, there is not enough information, we need more
- ☐ I don’t want more information
